# Evolutionary dynamics of nascent multicellular lineages

**DOI:** 10.1098/rspb.2024.1195

**Published:** 2025-04-30

**Authors:** Guilhem Doulcier, Philippe Remigi, Daniel Rexin, Paul B. Rainey

**Affiliations:** ^1^ Department of Philosophy, Macquarie University, Sydney, New SouthWales 2109, Australia; ^2^ Department ofTheoretical Biology, Max Planck Institute for Evolutionary Biology, Plön, Germany; ^3^ Department of Microbial Population Biology, Max Planck Institute for Evolutionary Biology, Plön, Germany; ^4^ Laboratory of Biophysics and Evolution, ESPCI, Université Paris Sciences et Lettres, Paris, France; ^5^ NewZealand Institute for Advanced Study, Auckland, New Zealand; ^6^ Laboratoire des Interactions Plantes-Microbes Environnement (LIPME), INRAE, CNRS, Université deToulouse, Castanet-Tolosan, France; ^7^ Institute of Environmental Science and Research Ltd., ESR, Porirua, New Zealand

**Keywords:** experimental evolution, Bayesian network, genealogies, evolutionary transitions in individuality, ecological scaffolding, multicellularity, metapopulations

## Abstract

The evolution of multicellular organisms involves the emergence of cellular collectives that eventually become units of selection in their own right. The process can be facilitated by ecological conditions that impose heritable variance in fitness on nascent collectives, with long-term persistence depending on the capacity of competing lineages to transition reliably between soma- and germ-like stages of proto-life cycles. Prior work with experimental bacterial populations showed rapid increases in collective-level fitness, with the capacity to switch between life cycle phases being a particular focus of selection. Here, we report experiments in which the most successful lineage from the earlier study was further propagated for 10 life cycle generations under regimes that required different investments in the soma-like phase. To explore the adaptive significance of switching, a control was included in which reliable transitioning between life cycle phases was abolished. The switch proved central to the maintenance of fitness. Moreover, in a non-switch treatment, where solutions to producing a robust and enduring soma-phase were required, the evolution of *mutL*-dependent switching emerged de novo. A newly developed computational pipeline (colgen) was used to display the moment-by-moment evolutionary dynamics of lineages, providing rare visual evidence of the roles of chance, history and selection. Colgen, underpinned by a Bayesian model, was further used to propagate hundreds of mutations back through temporal genealogical series, predict lineages and time points corresponding to changes of likely adaptive significance, and in one instance, via a combination of targeted sequencing, genetics and analyses of fitness consequences, the adaptive significance of a single mutation was demonstrated. Overall, our results shed light on the mechanisms by which collectives adapt to new selective challenges and demonstrate the value of genealogy-centred approaches for investigating the dynamics of lineage-level selection.

## 1. Introduction

Major evolutionary transitions in individuality underpin the emergence of life’s complexity [1–4]. A transition of particular interest is that involving the formation of multicellular collectives from ancestral unicellular types [5–8]. The transition initiates with the formation of simple groups—for example, through failure of cells to separate upon cell division—and completes once groups become units of selection in their own right. For the latter, newly formed collectives must be Darwinian; that is, collectives must be discrete (with variation among collectives), replicate and leave offspring copies that resemble parental types [9].

As elaborated elsewhere, these Darwinian properties are derived traits and require evolutionary explanation [10,11], but how these properties evolve—in the absence of collectives already manifesting these properties—has been a puzzle. One solution that has motivated experimental studies recognizes that variation, replication and heredity can be exogenously imposed (scaffolded) by specific ecological conditions [12–16]. Once stably imposed, simple undifferentiated groups of cells can become unwitting players in a selective process that occurs over timescales defined by the birth and death of groups, just as if they were cells of a multicellular organism.

A conceivable ecological setting—and one that has inspired laboratory studies—is provided by reeds in a pond [12]. Reeds act as scaffolds around which microbial mats attach, allowing colonization of the air–liquid interface (ALI), but importantly, spatial separation of reeds ensures that mats are discrete, thus allowing variation to manifest at the level of mats. Periodically, mats fail and sink, opening niches for the formation of new mats. If dispersing cells from an extant mat re-colonize a vacant reed, then the newly formed mat, being derived from the extant parent, may inherit parental characteristics. Mats thus become units of selection in their own right, with selection operating over two timescales: the doubling time of mats and the doubling time of cells [13]. Knowledge of the evolutionary dynamics that unfold at the level of mats stands to shed light on processes underpinning the origin of multicellular life.

In the laboratory, an analogy of the reeds-in-pond model has been devised by substituting glass vials for reeds with experimental realization in earlier studies [17,18]. In brief, the work uses the aerobic bacterium *Pseudomonas fluorescens* SBW25, whose metabolic activities, when introduced into static broth microcosms, cause the medium to become anoxic [19]. This creates conditions that favour cellulose-overproducing, ALI-colonizing (mat-forming) mutants known as ‘wrinkly spreaders’ (WS). Presence at the ALI ensures that constituent cells benefit from freely available oxygen. Given a cost associated with the overproduction of cellulose, WS mats are susceptible to invasion by cellulose-defective ‘smooth’ (SM) mutants. These types pay none of the costs but fail to provide structural support to the mat, ultimately leading to mat collapse [20]. While this amounts to a classic tragedy of the commons [21], taken back into the pond setting, functional roles of WS mat-forming cells and swimming SM cells can be viewed as analogous to primitive soma and germ phases, respectively [11]. WS mats perform an ecological role (ensuring access to oxygen) while producing seeds (SM cells) of the next generation of mats. The latter is especially relevant because in the absence of propagules, mats, like soma, are an evolutionary dead end [17].

**Figure 1.**
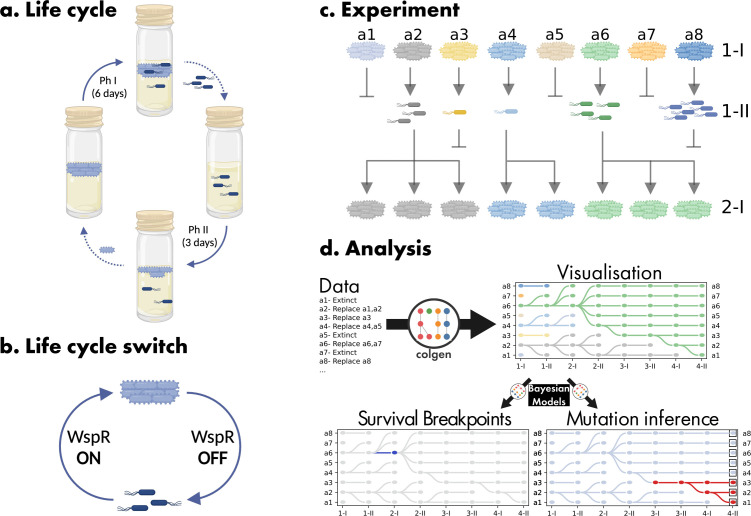
Experimental regime and colgen-based representation. (a) A single generation of the nascent multicellular life cycle has two phases. Phase I (Ph I) initiates with the placement of a single WS genotype into a glass microcosm. The WS type overproduces cellulose and forms a soma-like structure (a mat) at the air–liquid interface (ALI). During the 6-day Ph I stage, the mat, in addition to remaining at the ALI, must generate non-cellulose-producing (SM) cells that function as seeds (germ cells) for the next generation of mats (solid arrow). At the end of Ph I, SM germ cells are transferred to a fresh microcosm (dotted arrow) where the soma state must be re-established. After 3 days in phase II (Ph II), WS cells are collected, and a single genotype is used to found the next generation. Alternative experimental set-ups are described and contrasted in [17,18]. (b) Transition between soma (WS) and germ (SM) states is required for survival and involves activation and deactivation of cellulose production, which is affected by mutations that activate and deactivate the synthesis of cyclic-di-GMP. Of particular relevance is the derived ‘life cycle switch’ lcs ^+^ genotype that has enhanced capacity to transition between soma and germ states because of a mutation in *mutS* that causes frameshifting to occur at high frequency in a tract of guanine (G) residues in the active site of the WspR-encoded diguanylate cyclase. When the reading frame is intact, the cellulose-overproducing soma state is achieved but gain or loss of a G-residue causes a frameshift, deactivation of WspR, leading to realization of the germ state. (c) Extinction of lineages occurs if there are no soma cells after 6 days of Ph I, no germ cells after 3 days in Ph II or if the mat collapses during Ph I (soma failure). The death of lineages allows a death–birth dynamic to unfold with lineages functioning as units of selection in their own right. A single meta-population of eight distinct lineages is shown. While lineages compete, they never mix. Extant lineages not only replace themselves but also have the possibility of reproducing on the death of a competing lineage. Selection rewards persistence, which depends on reliable transitioning between states. (d) Schematic description of three functions of the colgen package: visualization (section 0.1), survival breakpoint detection (section 0.2) and belief propagation (section 0.3).

A graphical representation of both the life cycle and the lineage selection regime is provided in figure 1. Negative frequency-dependent interaction among WS and SM cells generates a life cycle that, in appropriate ecological context, becomes the primary focus of selection. Important to note are the causes of lineage-level death–birth dynamics. Lineages go extinct whenever they fail to generate the next stage in the life cycle or because the mat phase collapses prematurely (soma failure). Given that the life cycle is fuelled by spontaneous mutation, lineages are particularly prone to failure, with lineage death providing opportunity for extant lineages (chosen at random) to export their reproductive success to new microcosms in precisely the same way as a mat that falls from a reed provides opportunity for competing mats to reproduce.

The experiment originally conducted by Hammerschmidt *et al.* [17] was performed over 10 life cycle generations with selection rewarding lineages that repeatedly transitioned through soma and germ phases. On the conclusion of the experiment, a single lineage (line 17) had swept to fixation. Detailed analysis showed that success was a consequence of collective-level benefits arising from a simple genetic switch. Of a numerous array of mutations, two were of special significance: one that caused the WspR diguanylate cyclase (DGC) to achieve a state of constitutive activation (thus, overproducing cyclic-di-guanosine monophosphate (cyclic-di-GMP)) and eliciting overproduction of cellulose) and a second, loss-of-function mutation in *mutS* (A1489C), a gene involved in methyl-directed mismatch repair. The latter, while causing an overall elevation in mutation rate, specifically increases frame-shifting in tracts of guanine (G) residues [22]. While tracts of Gs are rarely found in any genome, one—composed of seven G residues—happens to reside in the active site of the DGC domain of WspR. Given the defect in *mutS*, WspR gained the ability to switch rapidly between functional and non-functional states (owing to elevated rates of frame-shifting), thus ensuring reliable transitions between soma and germ phases [17].

Here, we took the adaptive genotype and an isogenic lineage in which the genetic switch was rendered inoperable and asked whether the genetic switch delivers longer-term evolutionary advantage. We also explored the extent to which the switch facilitates lineage-level responses to the challenge of growing in larger diameter microcosms requiring substantially greater investment in the soma phase. Underpinning our analyses is colgen, a newly developed analytical tool that provides a graphical representation of genealogies, a variety of means for exploring lineage dynamics and, supported by a Bayesian model, allows inferences as to branch points of likely adaptive significance. Further, when combined with DNA sequences from derived lineages, colgen predicts the sequence of causal mutational events. Subsequent genetic analyses support the value of these predictions.

## 2. Material and methods

### (a) Experimental evolution of collectives

The experimental protocol is as described previously [17] and depicted in figure 1a–c. The two genotypes used here, line 17 (hereafter Life Cycle Switch+, lcs^+^ ) and the isogenic variant in which the *mutS* A1489C was reverted to wild-type (hereafter Life Cycle Switch−, lcs ^−^), are derived from [17].

Both lcs ^+^ and lcs ^−^ genotypes were propagated through 10 life cycle generations in standard (S) 17 mm diameter microcosms and in parallel, in large (L) microcosms of 35 mm diameter, with 9.5 and 31.5 ml media, respectively, to keep the surface area to volume ratio constant. Two genotypes, multiplied by two sizes of microcosm, result in four experimental regimes, designated S-lcs^+^ , L-lcs^+^ , S-lcs ^−^ and L-lcs ^−^.

Ten life cycle generations were performed on 48 parallel cultures blocked as six meta-populations of eight lineages. As in the reeds-in-pond thought experiment, lineages that fail to complete the life cycle and go extinct provide an opportunity for viable collective lineages to reproduce. Upon the demise of a lineage, a viable type from the same block is chosen at random and allowed to replace the extinct type. In the case where all cultures from a given block go extinct at the same time, random cultures from viable blocks are used to replace the extinct lineages. The eight-microcosm sub-population structure is a practical consequence of the experimental set-up in eight-microcosm racks. It limits global competition between lineages, as sub-populations must be entirely extinct before being invaded by lineages from other meta-populations.

### (b) Measuring lineage-level fitness

The standard assay of fitness in experimental evolution is to determine the growth rate of derived genotypes in direct competition with ancestral types ([23], performed for this system in [17]). This captures two components of fitness: fecundity and viability. Here, however, this measure is inadequate. First, it is necessary to consider a timescale longer than one growth period within one microcosm [15]—survival probability over the timescale of collective generations is relevant for assessing collective-level adaptation. Second, standard measures capture two components of fitness: fecundity and viability. Observation of the genealogies of lineages led to the realization that the probability of avoiding extinction is a sound predictor of future evolutionary success [24]. In addition, viability (and not fertility) is the only component of fitness that depends on the genotype of collectives in this experimental set-up. Indeed, surviving collectives have an equal chance of reproduction (with the total number of offspring being set by the number of extinct competing lineages). Henceforth, measures of fitness were determined not by competition with an ancestral type but by measuring the number of lineage extinction events after replicating lineages of single test genotypes have gone through a further life cycle generation. Further details are available in the electronic supplementary material, note S1, including additional growth experiments that show a response to collective-level selection.

**Figure 2.**
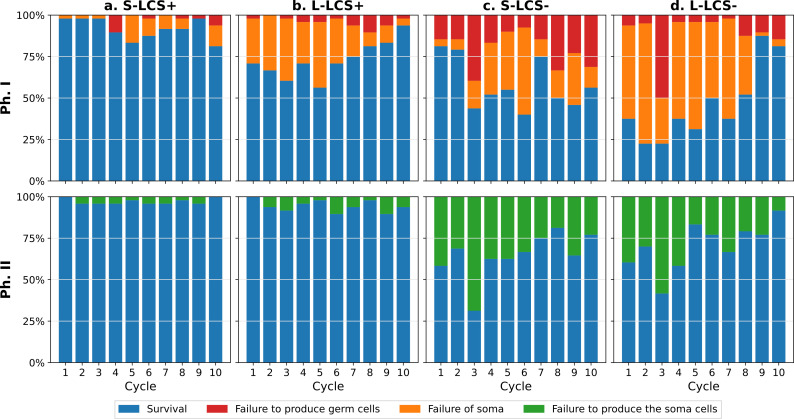
Proportion of surviving collectives in phase I (Ph I) and II (Ph II) for each cycle. The stacked bar corresponds to the proportion of surviving collective for each cycle. Blue denotes extant lineages, with red, orange and green marking extinct lineages and causes of extinction: during Ph I, failure to produce the germ cells (red), failure of the mat to remain intact for the required 6 days (orange), and during Ph II, failure to produce soma cells (green). Each column is a different treatment, lcs+: lineage 17 from [17] with a life cycle switch, lcs−: same lineage with the switch reverted, S: standard microcosms, L: large microcosms.

### (c) Sequencing

All WS genotypes chosen at the end of generation 10—in lieu of founding generation 11—were subject to whole genome sequencing. Bacterial genomic DNA was extracted from static overnight cultures of WS clones using the Wizard Genomic DNA purification kit (A1125, Promega) following the manufacturer’s instructions. Whole genome sequencing was performed using Illumina sequencing technology. Short reads were mapped on the *P. fluorescens* SBW25 reference genome (GCF000009225_2) and mutations were identified with Breseq v. 0.32.1 [25] using default parameters. When taking into account extinctions within cycle 10 and filtering out sequence data of poor quality, 45 of 48 endpoint genotypes were sequenced for L-lcs^+^ , 48 of 48 for S-lcs ^+^, 42 of 48 for L-lcs ^−^ and 37 of 48 for S-lcs ^−^. Further genotypes were targeted for sequencing using the same protocol after the experiment concluded, based on analysis of genealogies. In S-lcs^+^ , genotypes at each cycle, in L-lcs ^+^ 1-II-A7 (cycle 1, Ph II, lineage A7) 2-I-A5, 2-II-A6, 2-II-A7, 2-II-A8, 3-II-A1 and 3-II-E2 were sequenced in order to elucidate the origin of the *mutL* mutation. Further details are available in the electronic supplementary material, note S3.

### (d) Analysis of genealogies using the colgen package

Each new cycle of the experiment produced a set of complex data: for each lineage, the record of germ and soma-like cell census, the presence of the soma state, and additionally death–birth events were recorded. Plotting and visualization features of colgen [26], v. 2.0b1 were used to synthesize data without discounting complexity. Survival probability, as determined via colgen (section 0.2, described in the electronic supplementary material, note S2), was combined with DNA-sequence information to identify genotypes of likely adaptive significance (section 0.3, described in the electronic supplementary material, note S3).

All results obtained using colgen in the context of this manuscript can be explored interactively online:  https://zenodo.org/records/11170876 [27].

## 3. Results

### (a) Eco-evolutionary dynamics

Figure 2 shows the frequency of lineage extinctions and their causes. Extant lineages are in blue, with extinctions owing to failure to produce germ cells shown in red, failure of the soma phase (mat) in orange and failure to produce soma cells in green (see figure caption). The proportion of soma failure (i.e. mat collapse) is higher in large microcosms (L) than in standard microcosms (40 out of 96 versus 3 out of 96 in the first cycle). This shows that large microcosms present a new and more stringent challenge to lineages when compared to the ancestral environment of standard microcosms. lcs ^+^ treatments showed little change (S-lcs ^+^ even less than L-lcs^+^ ), which indicates that these lineages are close to an evolutionary equilibrium. By contrast, the proportion of lineages that survived in the lcs ^−^ treatments was initially low but rapidly increased. Figure 2 shows the proportion of lineages that avoided extinction at each of the 10 life cycle generations. It also reveals how changes in survival during one phase correlate with changes in survival during the other. Viewed as displayed in the electronic supplementary material, figure S1, and comparing the first and the tenth generations, the data indicate, for S-lcs ^−^ and L-lcs^+^ , a trade-off between survival in Ph I versus Ph II. For S-lcs^+^ , there is a decrease in Ph I survival that is not accompanied by an increase in PhII survival. However, PhII survival was already close to its maximal possible value, indicating the founding genotype was close to optimality, thus obscuring any trade-off. Finally, for L-lcs ^−^ no trade-off is seen, suggesting the series of mutations may have broken the trade-off [28].

Data presented in figure 2 average the proportion of collectives that avoid extinction across the entire population of lineages at each time point. It masks heterogeneity, including the fact that some lineages are evolutionary dead ends, while others rise to fixation. A more information-rich representation is one where the evolutionary fate of each lineage is displayed along with the causes of extinction.

**Figure 3.**
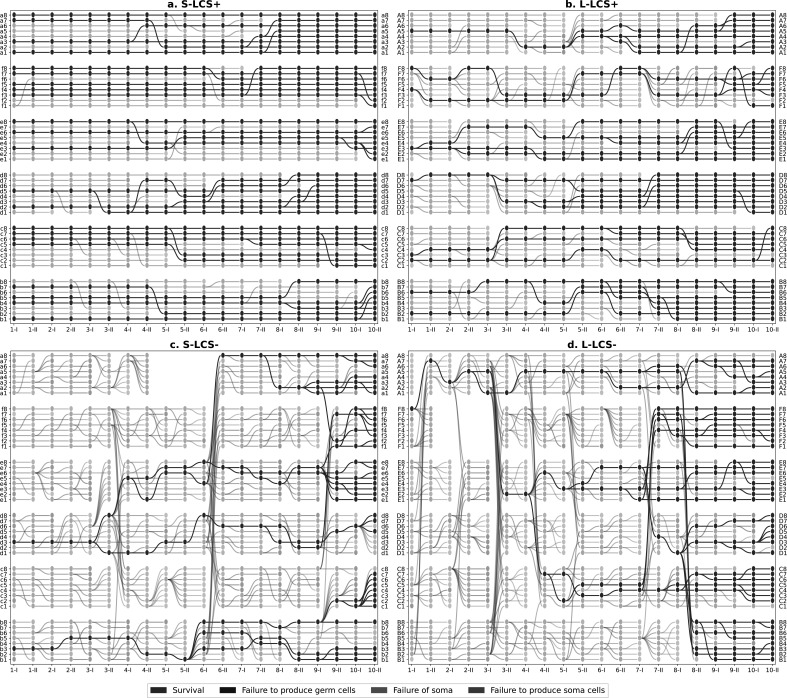
Lineage genealogies. The lineage-level selection experiment yields a rich dataset of 48 parallel microcosms (lineages) for each treatment with associated extinction (colours) and replacement events (edges). Lineage-level genealogies in the shape of directed acyclic graphs offer a synthetic but more granular visualization compared with the proportion of events at each phase (figure 2). The *x*-axis labels are phases within each life cycle generation, with 1-I indicating the end of Ph I of the first cycle, 1-II, the end of Ph II of the first cycle and so forth. The gaps (L-lcs ^+^ population F in 2-I, 2-II and 3-I, and S-lcs ^−^ population in 5-I, 5-II and 6-I) are missing owing to technical errors.

Figure 3 provides such a visual representation. Immediately apparent are treatment-specific differences in lineage dynamics, with those founded by lcs ^+^ suffering fewer extinction events compared with those founded by lcs ^−^. This is true for both continued selection in standard size microcosms and in the face of challenges posed by evolving in large diameter microcosms. Where improvement is evident, it is most apparent in the capacity of lineages to form mats able to endure the entire 6-day period of Ph I. Interestingly, this trend is reversed in S-lcs ^+^, where death owing to mat failure increased during the course of the experiment. Production of the germ-line phase during Ph I and the soma-phase during Ph II not only posed the greatest challenge to lcs ^−^ treatments but also showed improvement during the course of the experiment.

Examination of the coalescent tree(s) (depicted in solid colour) reveals further differences among treatments. lcs ^+^ maintained in standard microcosms showed few deaths, with half of the founding lineages remaining intact at the end of the experiment. On the other hand, lcs^−^ lineages propagated in large microcosms narrowly escaped extinction, with extant lineages, on conclusion of the experiment, being descendants of just a single ancestral microcosm (microcosm 2-II-A5—generation 2, Ph II, microcosm A5).

Collective fitness was assessed by estimating the probability of a collective to avoid extinction (See Material and Methods). The data, shown in the electronic supplementary material, figures S2 to S5, are largely concordant with that shown in figure 2. lcs ^+^ lineages evolving in standard microcosms showed an overall decline in fitness relative to the ancestral type (electronic supplementary material, figure S2a) with negative contributions coming from both reductions in durability of the soma phase (electronic supplementary material, figure S2b) and in capacity to switch between life cycle phases (electronic supplementary material, figure S2c,d). This negative trend was not apparent for lcs+ types in large microcosms, where the fitness of all derived lineages exceeds the ancestral type (electronic supplementary material, figure S3a).

The fitness of derived lcs ^−^ lineages in standard microcosms was highly variable, with some test genotypes having elevated fitness but others with reduced fitness. Low probability of switching was the primary cause of low fitness (electronic supplementary material, figure S4c,d). This is understandable in terms of the availability of mutational routes for switching between soma and germ phases becoming increasingly limited as a consequence of prior mutational cycles. By contrast, in large microcosms, there was a noticeable improvement in the fitness of all tested lcs ^−^ lineages (electronic supplementary material,figure S5a), primarily in terms of the ability to produce an enduring soma phase (electronic supplementary material, figure S5b).

### (b) Identifying likely adaptive events

The genealogical connections depicted in figure 3 show what appear to be selective sweeps. Those microcosms that mark the start of such sweeps indicate points at which adaptive mutations may have arisen. However, as the number of offspring at any time point is dependent on the death of competing lineages, the connection to adaptive potential can be difficult to infer. For example, it is not inconceivable that a poorly performing, but nonetheless extant, lineage happens to amplify, not because of improvements in fitness, but by chance. To distinguish improvements in future representation owing to chance versus selection, a measure of enduring ‘adaptive potential’ associated with each node is desirable.

To this end, we used a Bayesian network model in conjunction with an evidence-propagation algorithm [29] to predict improvement in the probability of survival. The model assumes the existence of a hidden *probability of survival* value for each node, which is calculated by computing the maximum-*a-posteriori* value using an expectation–maximization algorithm that takes genealogical data and transmission models as input. Details of the procedure are described in the electronic supplementary material, note S2. Nodes associated with marked improvement stand as indicators of time points at which mutations predicted to have adaptive significance occurred. While we bring particular focus to the L-lcs ^−^ treatment, identical analyses were performed on the remaining three treatments and are reported in the electronic supplementary material, figure S8.

**Figure 4.**
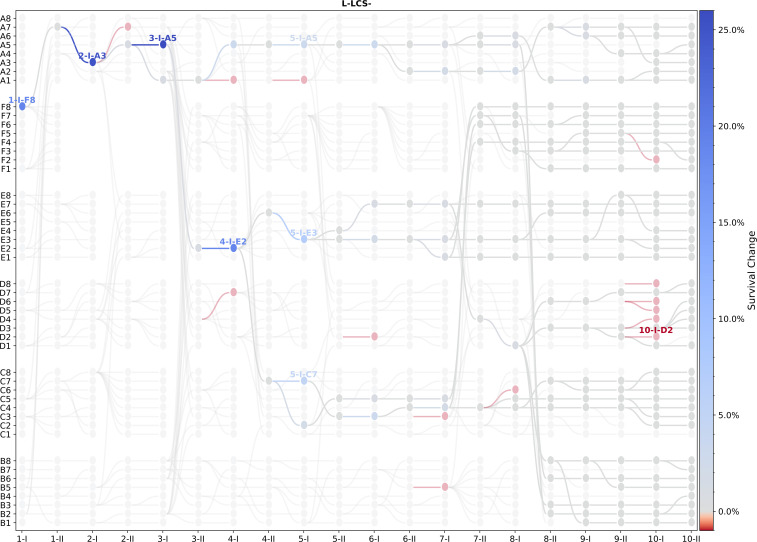
Nodes in the L-lcs ^−^ genealogy predicted to mark adaptive change. Lineages are coloured according to predicted change in survival probability relative to the previous cycle. The belief-propagation algorithm used for analysis is described in the electronic supplementary material, note S2. Here the model is beta with a grid of 100 points. The electronic supplementary material, figure S8 shows this result for all four treatments.

Figure 4 shows the result of the analysis for the L-lcs ^−^ treatment. According to the maximum-*a-posteriori* procedure, genotypes that are ancestors of lineages where the probability of survival markedly improved are 2-I-A3 (generation 2, Ph I, lineage A3; marked A3 on the figure, +25%), as well as 1-I-F8 (+19%), 2-II-A5 (+1%), 3-I-A5 (+26%), 3-II-E2 (+1%), 4-I-E2 (+19%), 5-I-E3 (+7%) and 5-I-C7 (+5%). Note that all these lineages had extant descendants at the end of the experiment. Moreover, 1-I-F8 and 2-I-A3 are ancestral to the entire population of lineages at generation 10.

In the next section, we describe how mutational data derived from whole genome sequencing—primarily of derived lines at generation 10—aid elucidation of the genetic bases of fitness improvements.

### (c) Propagating mutational data to the full genealogy

Whole genome sequence was obtained from all single WS (soma) genotypes derived from each extant lineage at the end of generation 10 (see the electronic supplementary material, Figure S13 for details), and from the single WS genotypes derived from the six selected microcosms marked in figure 4. DNA sequence reads were aligned to the ancestral SBW25 genome, leading to the identification of a total of 9271 mutations (across all four treatments). Because the 10 generations reported here were derived from lcs^+^ , which had already experienced 10 prior generations of evolution, we removed those mutations (58 in total) that accrued during the previous experiment (see the full dataset in the ‘Data availability’ section). While this represents an overwhelming number of mutations, in part, expected because of the mutator status of the lcs+ genotype, mapping of mutations on genealogies is nonetheless possible. A belief-propagation algorithm similar to that described in the previous section was used. This builds upon the fact that an identical mutation found in two or more descendant lineages most likely emerged in a single ancestral lineage whose identity can be inferred from patterns of genealogical connections. The procedure is detailed in the electronic supplementary material, note S3.

**Figure 5.**
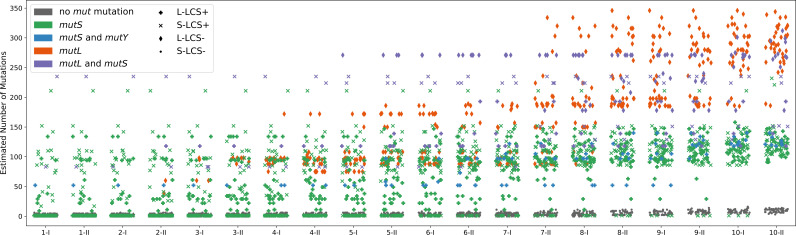
Reconstruction of the number of mutations through time. Each point is the number of new mutations (compared to the ancestor of the genealogy) predicted to be found in a single microcosm at a given phase in each cycle. The inference stems from propagating knowledge of mutations in the final derived genomes of extant lineages backwards through the genealogy using the belief-propagation algorithm described in the electronic supplementary material, note S3. The electronic supplementary material, figure S10 shows the number of identified mutations without reconstruction through the belief-propagation regime. The electronic supplementary material, figure S13 shows the number of mutations associated with each extant lineage (derived directly from genome sequence data).

Given the large number of mutations, analysis began by first propagating the mutations from the end point (combined with additional information from the six focal lineages from L-lcs ^−^) backwards through each genealogy. The results, shown in figure 5, reveal that lineages evolving under the S-lcs ^−^ treatment (represented by dots) accumulated few mutations during the course of the experiment. This is as expected given that a single mutation underpins the transition between each phase of the life cycle: two mutations per cycle and thus ≈20 over the course of 10 generations. By contrast, lineages from both lcs ^+^ treatments harboured ≈150 mutations, but some carried ≈250 mutations (represented by cross and plus symbols). Surprisingly, lineages from the L-lcs ^−^ treatment (represented by diamond symbols) harboured, for some lineages, more than 350 mutations.

Such large numbers of mutations are indicative of mutator genotypes. Mutation rate was not measured here; however, mutation rates of bacteria harbouring mutations in primary components of the methyl-directed mismatch repair (MMR) system have been previously measured and shown to be elevated by between 70- and 300-fold [30,31]. In addition, for non-mutator lineages in a similar experimental set-up, it is rare to fix more than a single mutation at each passage [31]. From the number of mutations found in final clones, we can infer that on average 29.0 mutations per collective generation are fixed for L-lcs ^−^, and respectively 12.2 mutations per generation for L-lcs^+^ , 13.0 mutations per generation for S-lcs ^+^ and 1.0 mutation per generation for S-lcs ^−^. Provided there are no changes in the probability of a mutation to fix, this estimate would indicate a 10- to 30-fold increase in mutation rate for mutator lines. That is, the same order of magnitude as reported in the literature.

While the presence of mutator genotypes was known for lcs ^+^ lineages, we nonetheless interrogated all mutational data for evidence of MMR-defective types (*mutS*, *mutL*, *mutY* and *mutM*). The points in figure 5, coloured according to genotype, show that microcosms harbouring lineages with large numbers of mutations also carry defects in mismatch repair genes (see the electronic supplementary material, table S1). Again, while known for the *mutS*-based lcs ^+^ lineages, these genotypes acquired additional mutations in MMR genes. For example, eight lineages in block A of lcs ^+^ evolving in large microcosms acquired a single non-synonymous change in the A/G-specific adenine glycosylase *mutY* (G153D). Interestingly, this *mutY* mutation did not generate more mutations (figure 5; electronic supplementary material, figure S15), but it is associated with a marked bias in the kind of mutations fixed (electronic supplementary material, figure S21).

**Figure 6.**
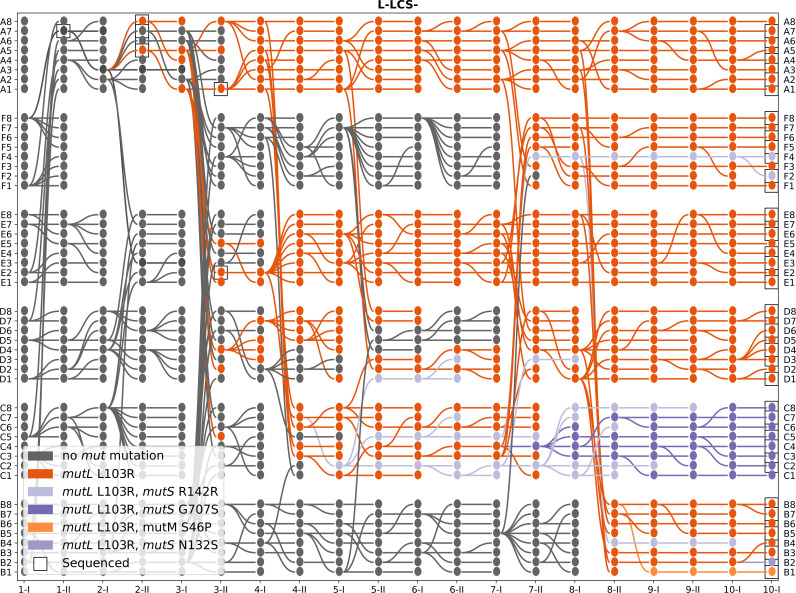
**
mutL** and **mutS** mutations in the L-lcs ^−^ genealogy. Lineages are coloured according to mutL and mutS mutations. The mutation mutL L103R appeared in lineage 2-II-A5 and swept to fixation. Black squares identify sequenced lineages, with the belief-propagation algorithm (described in the electronic supplementary material, note S3) allowing inferences to be extended to all nodes. The electronic supplementary material, figures S14 to S17 show the same analysis for all four treatments.

The greatest number of mutations, however, was found in lcs ^−^ lineages propagated in large microcosms. As more fully elaborated below, a single *mutL* (L103R) mutation (orange diamond symbols) arose early in the experiment, but seven lineages of block C also acquired an additional *mutS* (G707S) mutation (purple diamond symbols). Figure 6 and the electronic supplementary material, figures S14 to S16 show these mutations in the context of the genealogies apparent in each treatment.

As shown in figure 4, the node marking the greatest improvement in survival probability in L-lcs ^−^ is in the vicinity of 2-I-A3 and 2-II-A5. Additional sequencing of these nodes showed that the point of origin of *mutL* (L103R) was immediately prior to 2-II-A5 (see the electronic supplementary material, note S3e and figures S18 to S20 for details of the procedure and iterative use of the algorithm to incorporate new data).

### (d) Experimental confirmation of adaptive changes

The combined information provided by the survival probability inference (section 0.2) and sequencing-information propagation (section 0.3) points towards an adaptive change having occurred in (or in the vicinity of) microcosm 2-II-A5. In this section, we describe additional experiments conducted to test this hypothesis.

**Figure 7.**
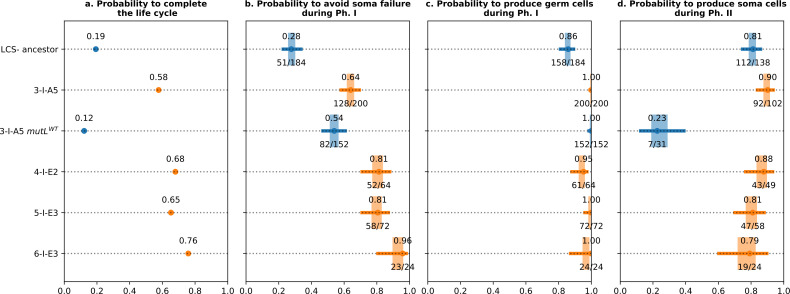
Probability and fitness estimates. Each line corresponds to a genotype derived from the ancestor lcs ^−^, and grown in large microcosms. Points correspond to the maximum-*a-posteriori* value for the probability; the fraction of surviving over the number of trials is written below the point. The box corresponds to the 50% Bayesian confidence interval, and the whiskers correspond to the 95% Bayesian confidence interval (the method is described in the electronic supplementary material, note S1). Orange depicts genotypes with the *mutL* L103R mutation; blue identifies genotypes without the *mutL* mutation.

Figure 7a–d shows the results of fitness assays performed around the adaptive points detected by Bayesian inference. These data, composed of measures of the probability to produce viable soma states at the end of Ph I (figure 7a), probability to transition from soma to germ during Ph I (figure 7b), the probability to switch from germ to soma type during Ph II (figure 7c), and overall life cycle fitness (figure 7d), confirm a stepped increase in life cycle fitness of the focal lineage between generation 2 and 4. Comparing the probabilities obtained through these additional experiments and the survival probabilities estimated by colgen shows agreement (electronic supplementary material, figure S9).

Because prior analysis predicted a causal role for the *mutL* (L103R) mutation, this mutation (in genotype 2-II-A5) was reverted, via a single nucleotide change, to the ancestral state. The effects of this change are shown in figure 7a–d. Data in figure 7d demonstrate that at the level of overall life cycle performance, the *mutL* (L103R) mutation is the primary cause of enhanced life cycle success. Interestingly, the effect is not significant for the two components of Ph I (figure 7a,b), but has a marked impact on the ability of the germ stage to switch to the soma state. Further visual evidence for a seminal role of the *mutL* mutation in switching between life cycle states comes from observation of germ cells arising within soma directly on agar plates (electronic supplementary material, figure S22).

## 4. Discussion

The evolution of multicellular life continues to receive attention from both empiricists and theoreticians [8,32–41]. In this work, using the experimental *Pseudomonas* system, we have explored the efficacy of a previously derived genetic switch controlling life cycle transitions, used knowledge of genealogical connections among nascent multicellular entities to predict adaptive events, combined these data with DNA sequence information, and, for one particular mutation, demonstrated a causal connection to improved fitness.

The genetic switch, which in a prior experiment had delivered adaptive benefit to competing lineages through significant improvement in control of expression of soma- and germ-like phases, proved, in this work, central to subsequent evolutionary success. This held not only for continued selection in standard microcosms but also for selection in large vessels. lcs+ lineages suffered few extinctions during further propagation in standard microcosms, whereas persistence of lcs ^−^ lineages in the same diameter microcosms proved challenging, with frequent death events caused by failures at all three life cycle phases.

Both lcs ^+^ and lcs ^−^ lineages struggled initially to find solutions to producing enduring mats in large-diameter microcosms, but in both instances, this capacity showed improvement during the course of the experiment. Notable in the L-lcs ^−^ treatment was the early discovery of a mutation in *mutL* that effectively converted lcs ^−^ to lcs^+^ . In the absence of this mutation—and thus marked improvement in capacity to transition between phases of the life cycle—all lineages in the L-lcs ^−^ treatment would have gone extinct by the third generation (evident by excluding reproduction events in figure 3 and following the fate of lineages to the first extinction). Thus, mutator-fuelled switching increased the reliability of the transition between life cycle phases, but the elevated mutation rate probably also played a role in the discovery of genetic changes that led to the formation of mats not only capable of colonizing the surface of large-diameter microcosms but with sufficient structure to ensure persistence over the 6 days of Ph I.

The prevalence of mutators associated with successful lineages was particularly apparent for lineages evolving in large microcosms and shows that the selective regime strongly favours mutational-based genetic switches. These switches deliver benefit through subsequent rounds of selection, despite presumed costs arising from the inevitable load of deleterious mutations. However, there are several reasons to suspect that these costs may be less significant than in standard mutation accumulation experiments. One important factor is the fact that the bottleneck is *selective,* with only cells expressing the correct phenotype transitioning to the next phase [42]. Such selective bottlenecks are likely to purge some deleterious mutations, thus reducing the mutational burden usually associated with hypermutagenesis [43]. Additionally, the 6- and 3-day periods of Ph I and II, respectively, provide ample time for both purifying selection and compensatory evolution. Finally, collective-level selection stands to counteract the accumulation of deleterious mutational effects: should a lineage carrying a costly mutation pass through the bottleneck such that it is unable to produce the next phenotype of the cycle, then its failure is compensated by replacement from the pool of extant lineages. Note that mutations which reduce growth rate, and thus appear maladaptive on an individual cell basis, can prove adaptive at the collective level [13] and over longer time scales [28].

While L-lcs ^+^ lineages were founded by a genotype carrying a *mutS* allele, a further *mutY* G153D mutation arose early in a single block A lineage and went to fixation by generation 10. That the mutation swept suggests that it may have contributed to further improvements in the survival probability of collectives (electronic supplementary material, figure S8); however, it is not inconceivable that *mutY* G153D hitchhiked with a mutation that improved the performance of the soma phase. Interestingly, this mutation did not add to the overall number of mutations (figure 5; electronic supplementary material, figure S15), but it did introduce a marked bias in the kinds of mutation found in these lineages (electronic supplementary material, figure S21), with recent work suggesting that the ensuing transversion-biased mutational spectrum might afford greater possibility for discovery of adaptive mutations [44]. Interrogation of sequence data for changes in repeat-tract length in genes with predicted DGC or phosphodiesterase (PDE) activity did not identify obvious candidate effectors of phenotype switching (as previously observed in WspR [17]; see figure 1).

In large microcosms founded by the lcs ^−^ lineage, in addition to the early and key *mutL* mutation, a further *mutS* G707S mutation arose in block C microcosms. As with the *mutY* allele arising in the *mutS* background, the *mutS* G707S mutation, although arising late in the experiment (at generation 7), fixed (in block C) within a further two generations (electronic supplementary material, figure S14). While the adaptive significance of this mutation was not further investigated, microcosm 8-I-C4 was identified by colgen as a point that marked significant improvement in survival probability (figure 4). Moreover, multilocus mutators have been shown to provide additional benefits to populations evolving under strong selection [45].

The fact that no additional mutators evolved in either the S-lcs ^−^ or the S-lcs ^+^ treatment suggests that additional opportunities for mutator alleles to hitchhike with beneficial mutations were provided in large microcosms. This makes intuitive sense given the combination of selective challenge, where founding genotypes were initially poorly adapted, a threefold increase in population size (compared to standard microcosms), and the fact that transitions through the life cycle require passage through single-cell bottlenecks. Under such conditions, there are probably numerous beneficial mutations upon which mutator alleles can hitchhike; moreover, selection is expected to favour cells that switch phenotype early because descendants of such cells have a heightened chance of passage through the bottleneck.

A notable difference in this study compared to previous work [17,18] is the mode of analysis, particularly that delivered by colgen. The first and arguably most valuable component of colgen is the simple visual representation of evolutionary dynamics via which evolutionary patterns previously invisible are revealed, allowing, for example, contributions of chance, history and selection to be directly observed. This is further aided by comparisons among the fates of lineages founded by lcs ^+^ and lcs ^−^ genotypes. The former being endowed with the adaptive Muts-WspR-based switch—the product of prior evolutionary history—whereas the latter, by virtue of reversion of the *mutS* allele to wild-type, has this component of history removed.

Lcs^+^ lineages propagated in standard microcosms suffered few extinction events (figure 3a), which can be directly attributed to prior evolutionary history and is consistent with the adaptive value of the switch. Notable was the worsening of performance after generation 3 (electronic supplementary material, Figure S2). This probably reflects the downside of few initial extinction events, resulting in a shift in the relative contributions of selection acting between- versus within-lineages [46]. In the absence of lineage-level death-birth events, selection operates primarily within lineages, leading to erosion of traits favouring the success of lineages. However, as lineage-level death-birth events occur, selection operates both within and between lineages, with, presumably, some equilibrium level ultimately being achieved.

By contrast, for lcs ^−^ lineages propagated in the same standard microcosms, multiple extinction events occurred (figure 3c). Beyond providing further evidence of the adaptive value of the switch, extinction and concomitant reproduction events are indicative of the hand of chance—both good and bad luck. In fact, as there is no overall improvement in the frequency of extinction events in S-lcs ^−^ lineages—and none detected by colgen (electronic supplementary material, figure S8), it is likely that the observed dynamics are driven almost entirely by chance.

Chance is an inherent factor in all evolutionary change, but a major factor affecting extinction events (figure 3). Chance underpins the ability to transition (by mutation) between life cycle phases, with mutational switches greatly improving the likelihood that transitions are successfully achieved, but chance also plays a role in mat durability, with subtle differences determining whether a mat endures the required 6 day period. Additionally, chance contributes to whether cells composing the next life cycle phase reach a frequency sufficient to ensure detection.

Extinction, while terminating the evolution of affected lineages, provides an opportunity for those that are extant to reproduce, and here again, chance is important. Death—often a consequence of bad luck—means that extant lineages, which may be equally susceptible to extinction, serendipitously gain further opportunity to explore genetic (and thus phenotypic) space, and through such exploration, the possibility presents that these lineages, at some future time point, might discover fitness-enhancing mutations: persistence, and its counterpart serendipity, is everything [24].

A single lineage from block B in the S-lcs ^−^ treatment is a case in point (figure 3c). The lineage in microcosm b3 produced offspring after the first generation, at generation 3 and at generation 4, but all failed soon after. Nonetheless, by virtue of persistence at generation 5, where the seven competing lineages failed, this lineage went on to leave eight offspring at generation 6; by the end of the experiment, this single generation 5 survivor had given rise to 11 extant offspring. Without further study, the extent to which the spread of this lineage reflects adaptive change is unclear, but it is plausible that success to this point was largely a matter of chance. This is also reflected in life cycle fitness data in the electronic supplementary material, figure S4, where there is no compelling evidence for the hand of selection, with the fitness of several extant lineages at the end of the experiment being equal to or worse than the ancestral type.

A further factor relevant to considerations that recognize the value of sheer persistence stems from the fact that lineage-level selection, as implemented in the experiments described here, involves a ‘death–birth’ dynamic. This is in contrast to a ‘birth–death’ process in which offspring replace extant collectives, regardless of fitness. While game theory approaches show that death-first dynamics favour the evolution of cooperation [47,48], in the context of this experiment a birth–death process would probably diminish the value of persistence.

Also notable in the dynamics of S-lcs ^−^ lineages (but also evident elsewhere) are abrupt extinctions of all lineages (within single blocks) following several generations of successful transitions (figure 3c). See, for example, blocks F and C at Ph I of generation 6. While possibly just chance, this more likely reflects deleterious effects of prior mutational history that, owing to lineages having traversed various mutational paths during prior cycles, have exhausted possibilities for realization of mutations critical for the next life cycle transition.

Turning to lcs ^+^ and lcs ^−^ lineages propagated in large microcosms, similar patterns manifest; however, also apparent are dynamics consistent with the hand of selection. lcs ^+^ lineages, although replete with genetic switch (a product of past history), suffered high levels of extinction during the first few generations, primarily owing to premature failure of the soma stage, with such events becoming increasingly rare from generation 5. While chance was dominant early on, and the effect of prior history minimal (as expected given that the founding genotypes had not previously experienced selection in large microcosms), sweeps arose from individual lineages, probably underpinned by adaptive change, resulting in the fixation of successful types by generation 10.

Lcs ^−^ lineages propagated in large microcosms, as described above, narrowly escape wholesale extinction. Having eliminated prior history through elimination of the genetic switch, the fate of these lineages was determined by both the requirement to find solutions to switching and the construction of robust mats. Again, as mentioned, a key adaptive change—a mutation in *mutL*—occurred at generation 2, with this lineage becoming the progenitor of all extant lineages (across all blocks) at generation 10. A combination of chance and selection was sufficient to overcome—and more than compensate for—prior evolutionary history.

Representation of genealogies via colgen, while invaluable in this work, is generally applicable to numerous kinds of lineage selection experiments where mixing of lineages is avoided, as in the ‘propagule-pool’ (as opposed to ‘migrant-pool’ or ‘mixed’) mode of reproduction [18,31,49]. This holds irrespective of whether collectives are clonal [17] or communities [14,50,51].

Beyond providing graphic visualization, colgen, by virtue of the underlying Bayesian model, has use in allowing mutational data obtained from the end of the experiment to be propagated backwards throughout the genealogy. This allowed sense to be made of the hundreds of mutations present in mutator lineages, which are typically overwhelmingly difficult to deal with. However, in addition, among the noise of mutations, colgen proved invaluable at identifying microcosms likely to contain adaptive mutations. Detailed investigations into L-lcs ^−^ led to the identification of the *mutL* mutation that was pivotal to the success of descendant lineages.

Graphical probabilistic models (such as Bayesian networks) for statistical inference have been used in haplotyping of pedigree [52], gene network inference [53] and genetic diseases risk assessment [54]. Bayesian network analysis presents several advantages. First, it is designed to work with partial data: inference can be performed even when sequence data are missing or partial. Second, such modes of analyses allow seamless integration of new data, with the possibility of adding new sequencing or fitness information to refine inferences. Thus, Bayesian analysis makes possible iterative protocols that alternate between inferences based on existing data and targeted new experimentation. Finally, the statistical model of observation and transmission of characters is relatively simple to define and can be adjusted for different purposes. While the models used above were minimal in their assumptions, it is possible to conceive and develop more complex mechanistic models as required.

It is evident that life cycles based on mutation can deliver benefits to nascent multicellular types. However, life cycles in extant multicellular organisms are typically affected via developmental mechanisms requiring changes in patterns of gene regulation. While it would be interesting to continue the propagation of lineages containing mutation-dependent switches to observe future refinement, there is minimal possibility that true developmental control could be achieved using an experimental protocol that requires soma and germ phases to express distinct morphologies on agar plates. This has led to a revision in which this requirement has been removed. Indeed, selection under a revised protocol that remains unchanged except for the fact that the germ-line phase is assessed by dispersal (and not colony morphology) has shown that lineages capable of transitioning between life cycle phases by developmental control are eminently achievable (Summers *et al*. in preparation).

## Data Availability

All the data used in this manuscript (genealogy records, mutation identification from read alignments, fitness assays) and the code necessary to reproduce all the figures from this dataset are available in the Zenodo repository: [55]. colgen v2.0b1 used in this manuscript is also available on Zenodo: [56]. Sequencing data is available in the European Nucleotide Archive accession number PRJEB79852. Supplementary material is available online [57].
